# Molecular epidemiology of Hepatitis C virus genotypes in Khyber Pakhtoonkhaw of Pakistan

**DOI:** 10.1186/1743-422X-7-203

**Published:** 2010-08-26

**Authors:** Amjad Ali, Habib Ahmed, Muhammad Idrees

**Affiliations:** 1 Deparment of Genetics, Hazara University, Garden Campus Mansehra Khyber Pakhtoonkhaw, Pakistan; 2 Division of Molecular Virology, National Centre of Excellence in Molecular Biology, 87-West Canal Bank Road Thokar Niaz Baig Lahore-53700, University of the Punjab Lahore, Pakistan

## Abstract

Six major Hepatitis C virus (HCV) genotypes and hundreds of subtypes have been identified globally. All these genotypes are generally studied for epidemiology, their vaccine development and clinical management. This article comments the frequency distribution of various HCV genotypes circulate in different areas/districts of Khyber Pakhtoonkhaw Province of Pakistan. Sum of 415 HCV RNA PCR positive sera samples were tested by a molecular genotyping assay. Data analysis revealed that out of these 415 HCV RNA positive patients 243 were males and 172 were females. Distribution breakup of the patients was 135, 58, 51, 51, 36, 32, 6, 7and 9 patients come from the districts of Abbottabad, Mardan, Pehawar, Swat, Haripure, Swabi and Dera Ismail Khan, respectively. Out of the tested samples, genotype specific PCR fragments were observed in 299 (74.82%) patient serum samples. The distribution of genotypes of the typeable samples was as fallows: 3 patients (0.72%) each were infected with genotype 1a and genotype 1b; 240 patients (80.26%) of genotype 3a; 25 patients (6.00%) genotype 3b; and 28 patients (6.73%) were observed as with mixed genotypic infection. Sums of 116 serum samples (27.88%) were still found untypeable by the used molecular genotyping system.

In conclusion, HCV genotypes 1a, 1b, 3a and 3b are distributed in various parts of KPK among which the genotype 3a is the most frequent genotype.

## Background

Hepatitis C virus (HCV) infection is accountable for the second most common cause of viral hepatitis and is one of the most important *Flaviviridae* infections with significant clinical problems all over the globe in humans [1]. At least six major HCV genotypes and hundreds of subtypes have been identified worldwide so far [2]. Dissimilar HCV genotypes are related to epidemiological studies, response rates to anti-viral treatment, vaccine development and clinical management of the infection [3]. HCV genotype is the strongest foretelling factor for sustained virological response since patients with different HCV genotypes act in response differently to alpha interferon therapy [4,5]. Solid evidence has been established that HCV genotype-2 and genotype-3 infected patents are more likely to have a sustained virological response (SVR) to anti-viral therapy than patients infected with genotype-1 HCV infections [6]. The reported rates of SVR to interferon plus ribavirin combination therapy are 65% and 30%, in patients infected with HCV-2/3 and HCV-1 genotypes respectively [7,8]. As the patient genotype has a vital role in treatment outcome therefore, should be done before starting standard interferon therapy.

Three HCV genotypes such as HCV-1, HCV-2, and HCV-3 have worldwide distribution and their relative prevalence varies from one geographic area to another. HCV-1a and 1b subtypes are the most prevailing genotypes circulating in the United States of America and Europe [4,9-11]. In Japan the most common circulating HCV subtype is 1b [12]. HCV-2a and 2b subtypes are mostly common in North America, Europe, and Japan and subtype 2c is found commonly in northern Italy [9-12]. HCV-4 is the most prevalent genotype circulating in North Africa and the Middle East [13,14]. HCV-5 and HCV-6 genotypes are establish only in South Africa and Hong Kong, respectively [15,16].

A small number of studies are available from Pakistan on the distribution of different hepatitis C virus genotypes only from the provinces of Punjab and Sindh [13,17-19]. No such study on the frequency distribution of various HCV genotypes and their modes of infectivity for different genotypes is available from Khyber Pakhtoonkhaw (KPK) of Pakistan. Therefore, this study was initiated to find out the molecular epidemiology of various HCV genotypes and subtypes present in KPK region of Pakistan and further to find out linked risk factors for its transmission.

## Methods

### Sampling

For the determination of HCV genotyping serum samples were collected along with specifically designed data sheets from patients admitted/attending various tertiary collection centers situated in different districts/parts of KPK, Pakistan. A written informed consent was taken from each patient. A printed data sheet was also filled for each patient contained demographic characteristic, possible mode of transmission, area/district, and estimated time of infection along with complete address and contact numbers of the patients.

### HCV RNA qualitative and quantitative PCRs

HCV RNA was detected qualitatively using reverse transcriptase (RT) PCR as described before [17]. Briefly, total RNA was isolated from 150 μl patient's sera samples using Gentra RNA isolation kit (Puregene, Minneapolis, MN 55441 USA) according to the kit protocol. Complimentary DNA (cDNA) of HCV 5'NCR was synthesized using 100 units of Moloney murine leukemia virus (MMLV) reverse transcriptase enzyme (RTEs) (Invitrogen, Corp., California USA) with 5 pM of outer antisense primer. Two rounds of PCR amplifications were done (first round PCR and Nested PCR) with two unites of Taq DNA polymerase enzyme (Invitrogen, Corp., California USA) in a volume of 2o μl reaction mix. The nested PCR products were run on 2% agarose gel contained ethidium bromide as DNA stain. The specific HCV PCR bands were visualized under UV transilluminator.

HCV RNA was quantified in all qualitative PCR positive sera using SmartCycler II Real-time PCR (Cepheid, Sunnyvale, Calif. USA) utilizing HCV RNA quantification kits (Sacace Biotechnologies, Italy). The SmartCycler II system is a PCR system by which amplification and detection were accomplished concurrently with TaqMan technology (Applied Biosystems, Foster City, Calif) using fluorescent probes to detect amplification after each replicating cycle. The lower and upper detection limits of the used assay were 5.0 × 10^2 ^and 5.0 × 10^8 ^IU/mL, respectively. Specimens yielding values above the upper limit were diluted 100-fold, retested and the obtained values were multiplied by this dilution factor to get the actual HCV RNA concentration in international units per mL.

### HCV Genotyping

For all the samples HCV genotyping was carried out using molecular HCV genotyping method previously published by Idrees [20]. Briefly, 10 μl (about 50 ng) of the extracted RNA was reverse transcribed to cDNA using 100 U of M-MLV RTEs at 37°C for 50 minutes. The RTEs were killed at 96°C for 5 minutes. Two μl of cDNA was used for the amplification of 470-bp region from HCV 5'NCR+Core region in first round PCR. Each first round PCR sample was subjected to two second-rounds nested PCR amplifications first with mix-A primers and the second with mix-B primers in a reaction volume of 20 μl. Mix-A had genotype-specific primers for 1a, 1b, 1c, 3a, 3c and 4 genotypes and mix-B contained genotype-specific primers for 2a, 2c, 3b, 5a, and 6a genotypes. The second round PCR products were electrophoresed on a 2% agarose gel to separate type-specific PCR fragment. The gel was stained with ethidium bromide and was observed under UV transilluminator. A 100-bp DNA ladder (Invitrogen, Corp., California, USA) was run in each gel as DNA size marker and the HCV genotype for each sample was determined by identifying the HCV genotype-specific PCR band. The gel photograph was taken using gel documentation system (Geldoc System, Eppendorf Inc, Germany).

### Statistical analysis

SPSS version 10.0 for windows was used for the analysis of data and summary statistics. The results for all variables were set in the form of rates (%). Fisher's exact and Chi Square tests were applied to find out the positive association among the categorical variables. The data was obtainable as mean values or number of patients. P-value less than 0.05 was considered as significant.

## Results

### Patients demographic

**Figure 1:**
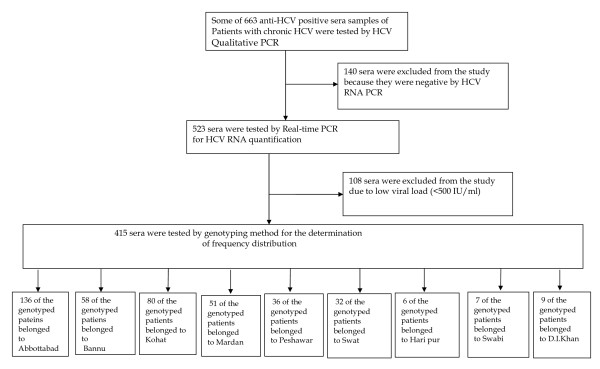
**Study disposition**. For study enrollments the patients were required to have chronic HCV with positive anti-HCV ELISA. The patients were also required to have detectable HCV RNA by qualitative RT-PCR and viral load >500 IU/ml and belonged to Khyber Pakhtoonkhaw province of Pakistan.

**Figure 2:**
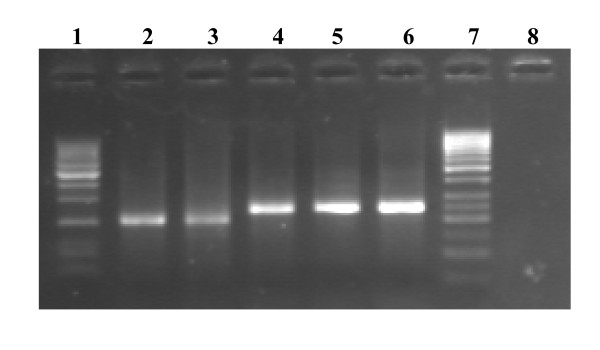
**Typical agarose gel electrophoresis patterns of PCR products from two different HCV genotypes**. Lanes 1 and 7 showing 50-bp DNA size ladder maker; Lanes 2-3 showed HCV-1a specific bands (210-bp); Lane 4-5 showed HCV-3a specific band (258-bp), Lane 6 showed Positive Control (258-bp HCV-3a genotype-specific band) and Lane 8 showed Negative Control (No band).

Results regarding the demographic distribution of HCV patients genotyped are summarized in figure 1. The figure also show enrolment and disposition criteria of the patients. The results revealed that out of total 663 anti-HCV positive sera that were received from different districts of KPK province, 523 were found positive by HCV qualitative PCR where as 140 sera samples were found negative by PCR and were thus excluded from further evaluation. Viral load was determined on all the 523 HCV RNA positive samples. In 108 samples the viral load was less than 500 IU/ml that is sensitivity of the genotyping assay, therefore, these low titer sera samples were excluded from the study for further genotyping analysis as were below the sensitivity limits of the genotyping assay. The selected 415 sera samples with moderate to high viral load (500 -5.0 × 10^8 ^IU/mL or above were tested by type-specific genotyping assay. The genotyped sera samples revealed that 136 belonged to Abbottabad region, 58 to Bannu, 80 to Kohat, 51 to Mardan, 36 to Peshawar, 32 to Swat, 6 to Haripur, 7 to Swabi and 9 patients came from D.I. khan. As all these serum samples that were included in the current study were tested HCV-RNA positive with enough viral load and could thus be genotyped by the utilized genotype-specific PCR assay. Figure-2 shows a typical agarose gel showing different HCV genotype-specific bands (HCV-1a & HCV-3a).

### Pattern of HCV genotypes in the study population

The distribution of HCV genotypes in the population analyzed is given in the table 1. The data shows that out of 415 tested sera samples, type-specific PCR fragments were seen in 299 (72.04%) whereas 116 (27.95%) sera samples were found untypable in the current study. The pattern of HCV genotypes of the typeable samples seen in the current study were in the order of: 240 (57.83%) were genotype 3a, 25 (6.02%) were with genotype 3b, 3 (0.72%) were 1a and 3 (0.72%) were 1b. where as sum 28 (6.73%) sera samples were infected with mixed genotype.

**Table 1:** Frequency distribution of HCV genotypes and subtypes in the studied population (N = 415)

HCV Genotype	HCV Subtype	No. of Isolates	Percentage
1	1a 1b	3 3	0.72 0.72
3	3a 3b	240 25	57.83 6.02
Mixed		28	6.73
Undetermined		116	27.95

Total		415	100

### Frequency distribution of HCV genotypes in different districts of KPK

Frequency distribution of different HCV genotypes were recorded from individuals belonged to various districts of KPK is shown in table 2. Among the determined genotypes 136 patients were from Abbottabad. Among the genotyped samples from Abbottabad, 83 (61.02%) belonged to genotype 3a, 5 (3.67%) were genotype 3b, 11 (8.08%) patients were infected by mixed genotype and 37 (27.02%) patients were observed as of unknown genotype. From Bannu HCV positive cases were 58, among these 32 (55.17%) were genotype 3a, 5 (8.62%) 3b, 3 (5.17%) were of dual genotype i.e 3a/1b. Sum 18 (31.03%) patients were of unknown genotype from District Bannu. Among 9 patients from D. I. Khan, 6 (66.66%) belonged to 3a genotype, 3 (33.33%) patients were observed as untypeable. Total 80 patients were positive to the corresponding virus from region of Kohat, 1 (1.25%) patient each from 1a and 1b genotype, 39 (48.75%) were of 3a genotype, 2 (2.5%) 3b genotype, mixed genotype was 6 (7.5%). Patients having unknown HCV genotype were 31 (38.75%). Among 51 Mardans patients 1a was 1 (1.96%) and 1b were 2 (3.92%), 3a were 28 (54.90%), 3b were 8 (15.68%), 7 (13.72) were dual genotypes and 5 (9.80) were untypeable genotypes. Of the 36 isolates from Peshawar city, 26 (72.22%) were 3a, 3 (8.33%) were 3b, 7 (19.44%) were untypeable and none was observed as with dual genotype. Of the 32 positive sera samples isolated from district Swat, 1a genotype one (3.125%) was of 1a genotype, 21 (65.625%) 3a, 2 (6.25%) 3b, mixed genotype 1 (3.125%), 7 (19.44%) were of untypeable genotype. From Haripur subtype 3a were present in 5 (83.33%) patients and 1 (16.66%) sample was found untypeable. All the 7 patients' sera collected from Swabi were found with untypeable genotypes.

**Table 2:** Prevalence of HCV of comprise genotypes in different geographical regions of KPK of Pakistan

Geno-type	Sub-type	Isolated from Abbottabad	Isolated from Bannu	Isolated from Kohat	Isolated from Mardan	Isolated from Peshawar	Isolated from Swat	Isolated from Hari pur	Isolated from Swabi	Isolated from D.I. khan	P value
1	1a	0	0	1 (1.25)	1 (1.96%)	0	1 (3.125%)	0	0	0	NS
	1b	0	0	1 (1.25%)	2 (3.92%)	0	0	0	0	0	NS
3	3a	83 (61.02%)	32 (55.17%)	39(48.75%)	28 (54.90%)	26(2.22%)	21 (65.625%)	5 (3.33%)	0	6 (6.66%)	<0.05
	3b	5 (3.67%)	5 (8.62%)	2 (2.5%)	8 (15.68%)	3 (8.33%)	2 (6.25%)	0	0	0	< 0.05
Mixed		11(8.08%)	3 (5.17%)	6 (7.5%)	7 (13.72%)	0	1 (3.125%)	0	0	0	NS
Undetermined		37 (27.20%)	18 (31.03%)	31 (38.75%)	5 (9.80%)	7 (19.44%)	7 (21.375%)	1 (6.66%)	7 (100%)	3 (3.33%)	> 0.05

Total		136	58	80	51	36	32	6	7	9	

### Occurrence of HCV with mixed genotypes

Table 3 shows the prevalence of HCV mixed-genotype infections determined during the current study in different populations across KPK of Pakistan. Total 28 HCV isolates were found having two genotypes. Of these, 11 belonged to Abbottabad region, 3 to Bannu district, 6 to Kohat district, 7 to Mardan district and only one to Swat district. Fourteen of the HCV infection with mixed genotypes had HCV genotypes 3a and 3b followed by 3a + 1b that were 10 (35.71%), 3a + 1a in 3 (10.71%) and 3a + 2b in 1 (3.57%).

**Table 3:** Prevalence of HCV mixed genotypes in KPK, Pakistan

Mixed genotype	From Abbottabad	From Bannu	From DIK	From Kohat	From Mardan	From Peshawar	From Swat	From Haripur	From Swabi	N
3a+3b	8	0	0	4	2	0	0	0	0	14
3a+1a	2	0	0	0	0	0	1	0	0	3
3a+1b	0	3	0	2	5	0	0	0	0	10
3a+2b	1	0	0	0	0	0	0	0	0	1

Total	11	3	0	6	7	0	1	0	0	28

### Potential risk factors associated with the transmission of various genotypes

Various possible risk factors observed in the current study responsible for infection transmission with each HCV genotypes are given in table 4. Over all the probable modes of spread observed were: 58.1% due to multiple uses of needles especially syringes, 16.7% due to surgeries (both major and minor), 3.3% due to blood and blood products infectivity and in 23.1% patients the mode of spread was not known and therefore were sporadic. The foremost mode of contamination in patients with HCV genotype 3a and 3b was multiple use and re-use of needles/syringes that was 70% and 60% respectively. All the genotype 1a and about 75% 1b infected patients got their infection during surgeries. Sixty percent of the patients having dual infections were sporadic where the route of infectivity was unknown to them. Majority (58.1%) of untypable patients were infected due to contaminated needles and syringes followed by surgeries and dental procedures.

**Table 4:** Potential routes of transmission of various HCV genotypes

HCV	Possible routes of transmission			
**HCV** **subtypes(N)**	**Re use of needles syringes (%)**	**Surgery, dentil operation (%)**	**Blood Transfusion (%)**	**Unclassified** **(%)**

1a (3)	0	3 (100)	0	0
1b (3)	0	2 (75)	0	1 (25)
3a (240)	168 (70)	33 (13.8)	6 (2.5)	33 (13.8)
3b (25)	15 (60)	7 (28)	0	3 (12)
Mixed (28)	5 (17.9)	4 (14.3)	02 (7.1)	17 (60.7)
Undetermined (116)	53 (45.7)	21 (18.1)	0	42 (36.2)

**Total (415)**	**241 (58.1)**	**70 (16.7)**	**08 (1.9)**	**96 (23.13)**

## Discussion

Khyber Pakhtoonkhaw (KPK) previously known as the North-West Frontier Province (NWP) is situated in the North-western of Pakistan and is one of the four provinces of Pakistan. It borders Gilgit-Baldistan to the north-east, Aghanistan to the north-west, the Federal Administrative Tribal Areas (FATA) to the west and south, Azad Jammu & Kashmir to the east, Balochistan to the south and Punjab and the Islamabad Capital Territory to the south-east. KPK is the third most populous province of the country. The main ethnic group in the province is *Pakhtuns*, followed by a number of smaller ethnic groups most notably, the Hindkowans; therefore, in the current study we tried to determine the pattern of HCV genotype in this specific ethnic group-*Pakhtuns*. A recently published genotype-specific PCR-based method [20] with increased sensitivity and specificity was employed for HCV genotypes determination. The data presented here corresponds to the preceding studies, in which genotypes, sub-types and/or serotypes were determined [9,21-23]. Analysis of the data showed that genotype 3a is the predominant genotype circulating in patients with chronic hepatitis C. These findings verified results of the earlier studies from Pakistan [17-19] which have concluded that genotype 3a is the most prevalent HCV genotype in Pakistan. Similarly in India, the predominant HCV genotype is 3a [24,25]. Our finding regarding distribution of the genotype seems to be similar to the genotype pattern reported from other Far Asian country such as Nepal [26] but different from those in South Asian countries such as Japan [27], Thailand [28] and Vietnam where genotype 1 is the major HCV genotype circulating in their populations.

Our study led to several important findings. The first finding is incidence of HCV genotypes that confirms the findings of another study from this country [23]. The second important finding of the study was the isolation of 27% isolates that were undetermined as no genotype-specific PCR products were seen for these samples. All these 116 sera samples with indetermined genotypes were HCV-RNA positive by qualitative PCR and were with sufficient viral titer therefore might be genotyped by the utilized genotype-specific PCR assay. A recent study from other parts of Pakistan showed only 6% HCV infected sera samples with untypable genotypes by this molecular biology-based system [23]. The high rate of untypable results seen in the current study may be due to the reason that majority (more than eighty percent) of our untypable patients had received standard interferon plus ribavirin treatment in the past and were either non-responders or were relapsed thereafter. Why the previously treated patients are difficult to genotype with higher sensitivity using this molecular based genotyping assay is not known to us.

We were unable to isolate even a single HCV-4 genotype from any infected patient that is believed to be absent from Pakistan, and is the most prevalent HCV genotype in Middle East [13]. None of the patients of the current study was found infected by genotype 5a and 6a. The two genotypes are reported from South Africa and Hong Kong, respectively [15,16] and may be absent or very rare in this part of the world.

The distribution of HCV genotypes for this population was examined district wise in order to establish a base line for regional differences in HCV pattern in KPK. No regional difference with respect to HCV genotype distribution in all districts was observed where the most prevalent genotype is 3a. However, a difference was observed in district Swabi where all the isolates were found untypeable. All these isolates had high titer of HCV RNA and could thus be genotyped however; majority of these patients had a history of interferon treatment.

In the current study sum 28 isolates of HCV patients had two genotypes at a time in their blood. Majority of these (39%) were the residents of district Abbottabad region where blood transfusion is common in thalassaemic patients. More than half of our patients with dual infection had HCV genotypes 3a and 3b. Like other studies, the prevalence of HCV mixed-genotype infections was high in thalassaemic patients who had received multiple blood transfusions. The overall rate of HCV mixed-genotype infections was 6.7%, which is the same as reported recently by Idrees and Riazuddin [23] from other provinces of the country.

It has been recognized in the current study that different HCV genotypes might be associated with different transmission routes. For example genotype 3a appears to be prevalent among injection drug users and dual infection among thalassaemic patients who had received blood transfusion several times in life. It is believed that HCV-3a was introduced into North America and the United Kingdom with the widespread use of heroin in the 1960s [29]. For more than 58% of our patients the probable modes of transmission observed were multiple uses and re-uses of needles/syringes. In 16.7% patients it was due to surgeries (both major and minor), 3.3% due to blood and blood products contamination and in 23.1% patients the mode of contamination was not known and was sporadic. The dominant mode of contamination in patients with HCV genotype 3a and 3b was multiple and re-use of needles/syringes that was 70% and 60% respectively. All the genotype 1a and 75% 1b infected patients got their infection during surgeries. Sixty percent of the patients having dual infections were sporadic where the route of contamination was unknown to them. Majority (58.1%) of untypable patients were infected by contaminated needles and syringes followed by surgeries and dental procedures. In Pakistan HCV-3a is the most widespread genotype as been also observed in the current study. It is believed that this genotype is spread by medical practitioners like doctors, vaccination teams and other medical persons used non-disposable syringes for injections attended a number of patients in the past. Mass vaccination in the recent past in which un-sterilized syringes were used might have enhanced the infection rate in this country [23]. This type of practice is still common in the countryside especially in KPK province which needs effective check for minimizing the spread of HCV infection and the transmission of other communicable diseases.

The only limitation of this study is the detection of large number (27%) of samples with untypable genotypes. All these samples were HCV-RNA positive, had sufficient viral titer and therefore might be genotyped by sequencing method to designate the exact genotype, however, we were unable to sequence these samples due to lack of sequencing facility in our campus.

## Conclusion

We conclude that (i) HCV genotypes 1a, 1b, 3a and 3b are distributed in various parts of KPK (ii) genotype 3a is the most frequent genotype circulating in KPK (iii) Major mode of HCV transmission is multiple uses and re-uses of needles/syringes.

## Abbreviations

HCV: hepatitis C virus; M-MLV: Molony-murine leukemia virus; NWFP: North West frontier province; KPK: Khyber Pakhtoonkhaw; ABI: Applied Biosystem Inc.; RT-PCR: reverse transcriptase polymerase chain reaction; cDNA: complimentary DNA.

## Competing interests

The authors declare that they have no competing interests.

## Authors' contributions

HA conceived the study, participated in its design and coordination and gave a critical view of manuscript writing. AA collected epidemiological data, performed genotype analysis and analyzed the data statistically. MI helped AA in molecular genotyping assays and gave a critical view of manuscript writing and participated in data analysis. All the authors read and approved the final manuscript.
